# Impact of a novel teaching method based on feedback, activity, individuality and relevance on students’ learning

**DOI:** 10.5116/ijme.56e3.e7ab

**Published:** 2016-03-20

**Authors:** Ovie Edafe, William S. Brooks, Simone N. Laskar, Miles W. Benjamin, Philip Chan

**Affiliations:** 1 Sheffield Teaching Hospitals Foundation Trust, United Kingdom; 2 Kings Mill Hospital, United Kingdom; 3 The Medical School, University of Sheffield, United Kingdom

**Keywords:** Clinical teaching, small group teaching, clinical students, teaching model, transition period

## Abstract

**Objectives:**

This study examines the perceived impact of a novel clinical teaching method based on FAIR principles (feedback, activity, individuality and relevance) on students’ learning on clinical placement.

**Methods:**

This was a qualitative research study. Participants were third year and final year medical students attached to one UK vascular firm over a four-year period (N=108). Students were asked to write a reflective essay on how FAIRness approach differs from previous clinical placement, and its advantages and disadvantages. Essays were thematically analysed and globally rated (positive, negative or neutral) by two independent researchers.

**Results:**

Over 90% of essays reported positive experiences of feedback, activity, individuality and relevance model.  The model provided multifaceted feedback; active participation; longitudinal improvement; relevance to stage of learning and future goals; structured teaching; professional development; safe learning environment; consultant involvement in teaching. Students perceived preparation for tutorials to be time intensive for tutors/students; a lack of teaching on medical sciences and direct observation of performance; more than once weekly sessions would be beneficial; some issues with peer and public feedback, relevance to upcoming exam and large group sizes. Students described negative experiences of “standard” clinical teaching.

**Conclusions:**

Progressive teaching programmes based on the FAIRness principles, feedback, activity, individuality and relevance, could be used as a model to improve current undergraduate clinical teaching.

## Introduction

Medical students are exposed to clinical teachers for most of the later years of their course, and their experience of clinical teaching is far-reaching, particularly in determining later career trajectories. There have been a number of studies looking at excellence in clinical teaching.^1^^-^^5^ These have mostly used an essentialist approach, extracting characteristics common to well-regarded clinical teachers and their teaching; few have served as a guide to the inexperienced clinical teacher.

More populist approaches have included the one minute preceptor,^6^ and Harden’s description of FAIR,^7^ which is an acronym standing for feedback, activity, individualisation and relevance. We have adopted the FAIRness approach to construct a model of progressive, classroom-based tutorials based on students’ own work and described the generally positive effects on adaptation of students to hospital clerkships during their first ever clinical placement.^8^^, ^^9^

We published a previous study exploring the role of FAIRness model on the adaptation of clinical students on their first clinical placement.^9^ This study is a larger examination of the role of the FAIRness approach on students’ learning of medicine, and the clinical method in particular. The previous study looked at first placements, and the present study only includes students who had previous experience of other clinical placements; therefore there is no data that is common to both studies.

Participants were third-year and final-year medical students’ placements on a single hospital surgical ward for four to six weeks and their experience of a FAIRness based programme targeted at their particular needs at their stage; introductory clinical method for third-years, and more complex integration of knowledge, synopsis and oral presentation of cases for senior students.

Briefly, the teaching model that is examined here was composed of weekly tutorials and written patient clerkings.  Students were expected to submit two written clerkings weekly, which would cumulatively form the basis of their end of placement assessment.  Anonymised extracts from these clerkings would be critiqued by students in small groups, and presented to the larger group.  In later tutorials, students were asked to make an oral presentation of their case, with another student asked to function as a critic. A common theme was developed in each tutorial, and a different theme in subsequent tutorials. There was an expectation of week-to-week improvement

The aim of this study was to examine how this teaching model, based on FAIRness, affected students’ experience of learning on clinical placement.

## Methods

### Study design

This was a qualitative research study. We analysed qualitative data in the form of unstructured reflective feedback essays.

### Study participants

The participants were all early third-year and late final-year medical students attached to one vascular surgical firm at a UK University Teaching Hospital over a four-year period (spring 2009 – winter 2012).  All students received teaching according to FAIR principles under supervision from one consultant (PC). All students had prior experience of clinical teaching on other firms; therefore these students were different from those reported in our previous study of the first ever clinical placement.^9^ The University of Sheffield Research Ethics Committee granted ethics approval.

### Data collection method

At the end of their clinical attachment, students were asked to provide voluntary anonymous written feedback on teaching sessions designed around FAIR principles. Students were asked to write a reflective essay with the title: “How does FAIRness teaching differ from standard teaching on the clinical attachment and what are its advantages and disadvantages from your individual point of view?”  The essays were submitted at the student’s individual end of placement assessment interview, and not seen until after the interview. It was made clear that the content could not, and would not, influence this assessment. There was no word limit on the essays. All feedback essays that were submitted were analysed.

### Data analysis

Responses ranged from 72 to 1903 words length, with an average length of 418 words. A total number of 108 students submitted reflective essays over the four-year period. Only one student did not submit an essay. All 108 essays were transcribed verbatim and managed using NVivo 10 software.  One researcher (OE) read the first 50 essays for data familiarisation, identifying major themes and highlighting key words. The essays were re-read, this time systematically coding the sentences and paragraphs. Saturation was considered at the 60th essay as no further codes were arising from the data. The initial coding structure was developed and condensed into an initial thematic framework. A deliberate variant case search was undertaken of essays 61-108; these were read and only coded where new issues arose.  A second researcher (WB), familiar with the whole data set, independently analysed a 10% sample of essays (n=1 and every 10th essay). Independent verification of themes was achieved; OE and WB met in person to discuss their thematic frameworks, identify differences, finalise the specific research question, and agreed on a relatively descriptive final thematic framework.

In addition, two researchers (MWB, NL) independently rated students’ experiences of FAIR in comparison to previous clinical placement(s). Each reflective essay was globally rated as positive, neutral or negative overall. For each reflective essay the two independent global ratings were stratified as follows: both global ratings positive (clearly positive), one positive and other neutral (positive trend); both neutral (neutral); one neutral and other negative (negative trend), both negative (clearly negative). The presence of polar opposites (i.e. one positive and other negative) triggered a third person (WB) to review the essay. SPSS was used to calculate Cronbach’s Alpha intraclass correlation coefficient.

## Results

Table 1 and Table 2, show themes on the advantages and disadvantages of FAIRness respectively (student essay number in square brackets). Global ratings of the essays were as follows: clearly positive views, 80/108 (74.1%); trend towards positive views, 20/108 (18.5%); neutral views, 3/108 (2.8%), trend towards negative views, 4/108 (3.7%), clearly negative views, 1/108 (0.9%). There were no polar opposite ratings by the two researchers. The intraclass correlation coefficient was 0.384, p=0.006.

### Experiences of the FAIRness teaching firm

As we anticipated, students recognised most of the elements of FAIR. They appreciated the feedback-rich environment of the class, where they were able to learn from the selected examples of their own and their colleagues’ actual work. This represented a safe learning environment and encouraged the group to improve over a longitudinal time frame.

**Table 1:** Advantages of FAIRness

Themes	Description	Quotes
Multifaceted feedback	FAIRness provides regular; timely; detailed; constructive feedback; both individual and group feedback from peers and the tutor. Feedback is specific to each individual. The structured sessions and learning environment facilitates regular and constructive feedback.	“Immediate feedback from both the supervisor and your peers — receiving constructive feedback makes you aware of any gaps in your knowledge, and gives you a goal to work towards for the following session” [Essay No. 17]; ‘The level of feedback provided by FAIRness teaching is far and above any other teaching I have encountered” [56];“the organised and detailed nature of the feedback in the FAIRness sessions have helped me learn where I specifically was lacking” [Essay No. 52]
Active participation	Sessions were interactive (everyone took part), enjoyable, and there was clear awareness of expectations, which allowed students to take responsibility and direct their learning. It demands participation in clinical activities and facilitates integration into the ward team; overall enhancing the learning experience.	“Allowed a more interactive and comprehensive learning experience than a more traditional approach may have” [Essay No. 13]; “Ensured that integration into the ward team has been smoother than usual due to increased interaction with them and the patients” [Essay No. 13]
Longitudinal improvement	Successive sessions and feedback allows longitudinal improvement at both individual and group levels; students can reflect on previous sessions. Individuality encourages conscientiousness of one’s work.	“During successive sessions, the previous feedback is used to improve the learning and performance of the student and this is applied in the next session” [Essay No. 10]; “enables the group to progress together and allows for amending of the objectives to bring everyone, hopefully, to a closer standard” [Essay No. 4]; “Having weekly sessions allows continuity of learning and enables us to see improvements within the group over time which is extremely useful” [Essay No. 41]
Relevance	Teaching is specific to stage of learning and applicable to clinical practice and summative assessment. In addition, it is directed at future roles as doctors, reinforces clinical skills often neglected and raises awareness of good clinical teaching (a key component of a junior doctor’s role).	“The sessions have been based around what is required of us as an Fl. This has worked really well, as most of the sessions have focussed on our presentation skills, which is a key skill that a house officer must demonstrate on a daily basis” [Essay No. 17]; “This programme differs from standard clinical teaching in that it was very relevant to our stage in training” [Essay No. 25]; “As an added bonus the FAIRness program was also a good revision tool in the run up to the exams” [Essay No. 32] “I think that the teaching was very relevant to our future career as doctors, if not to the phase 2 exams. Medical students can get very caught up in the obsession with passing exams, forgetting slightly that we are learning skills that we will use our whole lives, whatever specialty we decide upon” [Essay No. 72]
Structured teaching	Thoroughly planned, dedicated weekly sessions provided direction and maintained motivation. Structure of sessions is linked to learning outcomes; this would ensure standardised regular teaching across the firm. The FAIR acronym aids structure.	“an obviously well thought out and crafted teaching method with undoubted applications for adult learning and undergraduate medical education” [Essay No. 1]; “I feel that the FAIR method is useful in providing a structure for clinical teaching sessions, and if adopted by every facilitator could be conducive to an improvement in the standard of medical student teaching” [Essay No. 11]; “The FAIRness principle is an effective structure to guide learning and maintain student motivation when on a firm” [Essay No. 38]
Professional development	Develop skills in critical thinking, delivering feedback and accepting criticism. Self-evaluation/critical assessment of work; assess strengths, weaknesses and progress against peers; encourages improvement to meet standards. Peer learning from observations of peers’ performance, peer feedback; collectively improve as a group.	“allowed us to begin to develop skills of peer review” [Essay No. 58]; “Being critical of your own work in this career is important. This is one of the challenges of these sessions in that you have to take the criticism for benefit and not let it get you down. In essence, getting over insecurities and being able to improve following the criticisms makes you a stronger student. The sessions are an invaluable experience in medical training” [Essay No. 53]; “I was able to listen to other peers and how they present but also critique what parts of the history were useful and which part not” [Essay No. 5]
Consultant facilitation	Facilitation by consultant interested in teaching; ensures quality; regular contact with consultant; better integration to clinical environment.	“the only time where I have had regular contact with a senior member of the medical profession who was able to give one-on-one advice on how to improve my history taking and examination skills” [Essay No. 31]; “The sessions also meant that I had a reasonable amount of time with my consultant which in other placements I have not had, this meant I got good feedback from a senior doctor and also meant I felt more included in the firm which hasn’t always been the case in previous placements” [Essay No. 49]
Safe learning environment	Small group teaching away from the ward; non-judgemental and honest environment; engagement with tutor.	“Fairness teaching gives a small group of students the opportunity to receive individualised feedback in an environment away from the glare or the ward and all its inhabitants” [Essay No. 14] ; “allowed an atmosphere of honesty to exist inside the session when students might normally be afraid of treading on the toes of the others” [Essay No. 76]; “The honest nature of the critique and the removal of “feel-good” comments are refreshing compared to the non-specific feedback usually given at other tutorials” [Essay No. 7]

**Table 2:** Disadvantages of FAIRness

Themes	Descriptions	Quotes
Time intensive	Time consuming for tutors, as it requires a lot of planning. Tutors would also need to be trained to deliver the sessions. In addition, students perceived sessions were too frequent, labour intensive and often too long (owing to large groups).	“Difficult to find teachers willing to commit to the time that the fairness model will take” [Essay No. 14]; “The main disadvantage of the FAIRness programme is that it requires more planning than standard clinical teaching, which is often improvised, and is generally more time intensive” [Essay No. 24]; “The preparation of histories and structuring them took quite a lot of time each week” [Essay No. 4] “One of the disadvantages of FAIRness is that it takes longer as time must be allocated for the student to have an active role, and for feedback to be given” [Essay No. 18]
Lacks specialist/medical science teaching	Lack of in depth teaching on medical sciences, clinical decision making such as investigations and management; all of which would be beneficial particularly with exams.	“If this program could be employed to give students some medical knowledge and to test it in a FAIR way, that would certainly help us a lot. If, however, this program exists only to test our history taking, I am not definitely sure how this will make us become doctors with a rounded outlook” [Essay No. 34]; “I feel more focus could have been given to the case in general (such as investigations, management etc.) to allow us all to get learning points from each case” [Essay No. 40]
Issues with feedback	Peer feedback is not always constructive or honest. Giving only private feedback may be better as it prevents embarrassment or intimidation if a student’s work was not up to standards of members in the group. Over emphasis on feedback and repetitive in large groups.	“Using peers meant that some students were intimidated and possibly were not as brutal, and therefore constructive, in their criticisms” [Essay No. 15]; “Though feedback encourages active learning, it should not be the sole contribution to this second component of FAIRness, for, in large groups, to get each student to deliver feedback is impracticable (too much repetition)” [Essay No. 1]
Lack of direct observation	Students’ interactions with patients were not directly observed on the ward and there was no bedside teaching.	“No direct observation of clinical history taking so quality of actual history cannot be ensured.” [Essay No. 37]; “it may not be representative of how the student is performing in the placement as a whole……the students are largely assessed through their individual performances on the written histories, which can produce a large amount of bias” [Essay No. 51]
Only once weekly sessions	Sessions may not be representative of student’s overall performance in the placement. More frequent sessions would widen focus of teaching to include other generic clinical skills.	“I believe that it would be even more beneficial if it were possible to have more sessions, and not just focus on clerking skills, but some of the other skills that we as student doctors need to develop” [Essay No. 36]
Relevance	Some sessions may not be relevant to upcoming exams; pitched beyond curriculum requirement and learning objectives (i.e. aimed at junior doctors). Simulation/artificial environment not the same as practising on the wards.	“Doing these full clerkings meant that each system examination was not done from beginning to end properly as would be required in the OSCE“ [Essay No. 78]; “the presentations of patients on the wards are often spontaneous, whereas many of the students pre-rehearsed and structured their presentations for the teaching sessions so it was not an accurate reflection of what would be required of them when they eventually become foundation year doctors” [Essay No. 78]
Large group	Group size too big in some cases which limits activity and engagement with sessions. In addition, feedback is repetitive and tedious in large groups. Large groups can be too intimidating for students to engage.	“I feel that the sessions would work better in smaller groups, as in a group of 14 the sessions took a very long time and sometimes felt a little repetitive” [Essay No. 47]; “The feedback provided was always useful and relevant, however due to this individualised approach with a single designated assessor and presenter, the group size and the number of histories to be presented led to some concentration issues among those not assessing or presenting” [Essay No. 76]

Students showed a preference for the active mode of learning and understood the relevance of the skills they were learning. They were less clear that the model allowed differences in individual rates and modes of learning; however they did recognise that there was individualised feedback. They felt confidence in the structure of the learning program, and appreciated that they were picking up lifelong skills additional to presentation of clerkings.

Students were also positive about elements outside the FAIR paradigm. The progressive longitudinal model of improvement was clearly recognised as beneficial. The continuous involvement of a senior consultant was seen to be unusual, but strongly positive.

### Experiences of other teaching firms

Although the main purpose of this study was to evaluate the FAIRness program in comparison to previous experiences of clinical teaching, many students made very strong comments on these previous experiences, which are worthy of note and reflection. Students described “standard” teaching as not being tailored to the need of the individual:

“it is often the case where one or two more motivated or experienced students get a lot out of a session at the expense of others, either because the teaching is pitched over the heads of some members of the group, or simply that some students are not as able to adapt themselves to a particular learning style or environment” [Essay No. 24].  

There were feelings of teaching not being relevant to stage of learning and being biased towards tutors’ preferences or specialty:

“consultant led teaching is often heavily biased towards their speciality or personal interest, which is often irrelevant to the learning objectives” [Essay No. 38];

“the really relevant skills of examination, history taking and basic communication are often overlooked and rarely taught” [Essay No. 48].

“Standard” teaching sounded passive. Students’ activity in pursuit of their learning tended to occur around their end of placement assessment. However, some placements provided interactive teaching:

“much of my teaching on my anaesthetic placement was based around small group sessions with much active learning, i.e. being asked questions on certain subjects” [Essay No. 28].

One student acknowledged factors that limit activity such as topic and time available for teaching:

“active learning can be difficult to incorporate into every means of teaching due to factors such as time constraints or topics of disinterest to students” [Essay No. 29].

There was a lack of consultant teaching, which, when provided, was perceived as often random:

“The [FAIRness] placement contrasted strongly with my previous rotation where consultant teaching was limited and haphazard” [Essay No. 37].

House officers are usually the source of teaching:

“Instead, teaching more commonly comes from the Junior House Officer who can cover a wider range of topics from history taking and examination to more specific aspects of the particular firm. However, these sessions are highly variable and dependent on the Fl (Foundation doctor year one) or the business of the ward” [Essay No. 44].

Students felt feedback had been generally minimal and poor quality:

“Feedback is not always received whilst undergoing standard clinical teaching on the wards, yet is invaluable in assisting the learning process” [Essay No. 20];

“a student would be very lucky to have their work individually analysed by a consultant. Even if their work was individually viewed, such as by presenting a case history on a ward round, there would be very little time for the relevant consultant to provide informative and constructive feedback” [Essay No. 51];

“Feedback is quickly becoming a major part of all clinical teaching. Typically this involves the student giving feedback on the teaching, rather than the student receiving feedback on how well they have demonstrated learning” [Essay No. 8].

Small group tutorials and bedside teaching combined with the use of feedback proformas were noted as good sources of feedback:

“Mini-CEX forms and DOPS forms are in place to allow assessors, particularly junior staff, to give guidance on areas of improvement for the student” [Essay No. 30].

Students were perceptive about the structure of their teaching and learning. They mentioned that standard teaching was not structured to achieve educational goals, consisting mostly of impromptu bedside teaching.

“From a personal point of view my clinical teaching has been very good in many areas but has not been structured in terms of long term goals, individualisation and review” [Essay No. 28];

“Even good teachers struggle if there is a lack of structure to what they are trying to achieve” [Essay No. 28];

“Standard clinical teaching, either by the bedside or in a small group situation, is often very variable in quality for several reasons. It is usually delivered in an impromptu way with little planning, and while some teachers can do this well, many teaching sessions are hindered by poor structure and no real aims” [Essay No. 24].

## Discussion

The use of standardised essays in qualitative research is well accepted.^10^^-^^12^ We chose to analyse the content of essays, as we felt this would give a more considered view than the traditional questionnaire or interview. Students are used to producing reflective pieces as part of their course; and having had time to reflect on both the merits and demerits of their clinical teaching, the responses may present some advantages over data gathered in more immediate face-to-face environments.

We took elaborate and explicit precautions to ensure the contents of these essays were not biased by external factors; all students had prior experience of teaching in other clinical environments, students were clear that honest opinions were sought, they would remain anonymous, and that they would not be penalised for negative comments. Indeed, negative feedback was purposefully sought by asking for “disadvantages” in the essay title.

In over 90% of essays, positive comments outweighed negative comments. Students genuinely appreciated structured and time set aside for dedicated teaching; with an emphasis on longitudinal improvement, both private and public feedback, as well as relevance to their future practice and the acquisition of generic skills. There are clear references to the acquisition of lifelong generic skills, which testify to the probable future impact of this form of teaching.

We have avoided evaluating the impact of this teaching on medical school exam results, as we do not feel this is relevant to the core purpose of this teaching. To confound matters further, this placement is often used by the medical school to monitor students who are experiencing difficulties with their studies; both as a remedial exercise and to utilise the closer observation of the students that the teaching method entails.

It is interesting to note that the most common negative comment (“disadvantage”) was about the program being difficult for the tutor, demanding greater commitment than most tutors would be willing to make. This view is coloured by students’ experience of previous tutors; but need not apply to new cohorts of suitably trained, professional tutors.

As expected, there were negative comments on what these tutorials were not intended for. They were never set up for coaching individual skills by direct observation, or for cramming for examinations. Unexpectedly, feedback came in for both favourable and unfavourable comment; although the authors have never before encountered a complaint of “too much feedback.” It was the express intention of this instructional design to create a feedback-rich environment, in which students could experience feedback on their individual performance; mostly by written tutor comments and self-appraisal (internally comparing their work with the work being actively critiqued by the group), and less often by having their work selected for direct appraisal by the group. Engaging students in providing critical feedback was also intended so that they could apply this skill to their own work.

Students’ reflection on the difference between standard clinical teaching and the FAIRness model was perceptive and thought-provoking. The Individualisation component of FAIRness is sometimes the most difficult for a teacher to understand and incorporate into their teaching. Students did feel that the FAIRness sessions had an aspect of personalisation within them; probably related to regular marking of their work, as well as selection of authentic work for tutorials.

We were a little shocked at the negative perceptions of standard clinical teaching in the students’ comparisons. In our previous work with first-placement student groups, (none of whom featured in the current work) we noticed a very negative perception of clinical teaching even before they had started their very first clinical placement; clearly, the opinions do not improve with time and experience.^3^ Students’ perceptions of “standard” clinical teaching paints a picture of haphazard, unplanned, passive sessions, with low involvement of senior teachers, poor opportunities for feedback on individual performance, and no real opportunities to have improvement noted and certified.  These experiences would have been gained in several centres around the central teaching hospitals; and we have no reason to believe that Sheffield is worse than any other teaching centre in this regard.

Many of these deficiencies have been described before, both in the UK and elsewhere,^3^^,^^6^ and might be regarded as generic weaknesses of our current organisation of clinical medical education. If this study is a true picture of the current state of undergraduate clinical teaching, then this represents both a major condemnation of our stewardship of undergraduate clinical medical education, and a huge challenge for the years ahead. 

### Limitations of the study

There are number of limitations to this study. All students were from a single medical school, and although clinical teaching does not differ much among UK medical schools, the students’ perceptions may not be generalisable to other medical schools.

The primary data is an essay written at the end of the FAIRness placement, where the memories of their recent experiences would be more immediate than the memories of comparative experiences on other placements. It is unclear whether this systematic difference would influence the students’ perceptions positively or negatively in either direction. The semi-structured format may additionally have limited the richness of data.

## Conclusions

The advantages offered by progressive programmes rooted in FAIRness principles could assist as a model of improvement for clinical teaching.

### Conflict of Interest

The authors declare that they have no conflict of interest.
